# IL-25 Promotes Th2 Immunity Responses in Asthmatic Mice via Nuocytes Activation

**DOI:** 10.1371/journal.pone.0162393

**Published:** 2016-09-12

**Authors:** Chao Wang, Qingfa Liu, Fangfang Chen, Wenjuan Xu, Caiqing Zhang, Wei Xiao

**Affiliations:** 1 Department of Respiratory Medicine, Shandong Provincial Qianfoshan Hospital, Shandong University, Jinan, Shandong, China; 2 School of Medical Sciences, Shandong University, Jinan, Shandong, China; 3 Department of Respiratory Medicine, Qilu Hospital of Shandong University, Jinan, Shandong, China; East Carolina University, UNITED STATES

## Abstract

**Background:**

Interleukin-25 (*IL-25*) is a potent activator of type-2 immune responses, and is responsible for airway inflammation in asthma. Previous reports have shown that *IL-25* expressed hyper-reactivity in an experimental mouse-model of asthma. In addition, the production of *IL-13/IL-5* promoted by nuocytes induced airway inflammation. Thus, it has been questioned whether blocking *IL-25* against its receptor *IL-17BR* could inhibit the expression of *IL-13* and *IL-5* via nuocytes, and further protect against inflammation in ovalbumin (OVA) induced mouse-model of asthma.

**Methods:**

In this study, in order to investigate the correlation among *IL-25*, *IL-5*, *IL-13* and nuocyte activities, we used OVA-sensitization and -challenging to induce the mouse model of asthma. The murine asthmatic model was validated by histology. The expressions of *IL-5*, *IL-13* and *IL-25* were detected by ELISA, quantitative real-time PCR, and western blotting of the lung tissue. Nuocyte activation was identified by the levels of *ICOS* (clone C398.4A) and *T1/ST2* (cloneDJ8) (acting as nuocytes surface markers) in the bronchoalveolar lavage fluid (BALF). This, in turn, was done by means of flow cytometry. The expressions of *IL-25*, *IL-5* and *IL-13* in our murine model were detected in the BALF.

**Results:**

The mice sensitized and challenged with OVA showed a high expression of *IL-25* in both the mRNA and protein levels in lungs. The expressions of *ICOS* and *T1/ST2* in BALF were increased. A significant correlation between *IL-25* mRNA, protein, and other Th2-cell producing cytokines (such as *IL-5* and *IL-13*) moreover were identified. Furthermore, when the asthmatic mice were treated with anti-*IL-25*, both the inflammatory cells’ infiltration and the inflammatory cytokines’ secretion were significantly decreased. The present findings indicate that *IL-25* might be involved in a series of asthmatic immune responses, playing an important role in the increase of nuocytes, and that its activation is necessary in maintaining Th2 central memory and sustaining asthmatic inflammation.

**Conclusion:**

This study showed that *IL-25* promoted the accumulation of *ICOS* and *T1/ST2* on nuocytes, further induced the pro-inflammatory Th2 cells, and promoted Th2 cytokine responses in OVA-induced airway inflammation.

## Introduction

Allergic asthma is airway inflammation accompanied by an increased secretion of cytokines. The infiltration of eosinophils, excessive mucus production, and airway hyper-reactivity induces airway narrowing and significant morbidity [1]. Many cytokines and chemokines, especially those ones derived from T helper type-2 (Th2) cells, play an important role in the pathophysiology of asthma [2].

Th2 immune-response aims to destroy pathogens that occur outside cells (bacteria and parasites) through one of the main types of helper T cells–Th2 cells. Th2 cells produce several Th2-type cytokines including interleukins 4 *(IL-4*), 5 (*IL-5*), 13 (*IL-13*), and 9 (*IL-9*), which all drive asthmatic pathology [3]. Among these interleukins, *IL-13* is secreted by Th2 cells in particular, and it is a mediator of allergic inflammation and disease. *IL-5* is a key mediator in eosinophil activations, and can stimulate B cell growth and increase immunoglobulin by binding to the *IL-5* receptor. Interleukin-25 (*IL-25*), also known as Interleukin-17E (*IL-17E*) can promote type-2 (Th2) immune responses [4]. Thus, it has been demonstrated that IL-25 can induce both *IL-5* and *IL-13* in various mouse cell lines [5]. However, the detailed mechanism of how *IL-25* induces Th2 inflammatory cytokines, such as *IL-5* and *IL-13*, is still unclear.

Recent studies have detected the expression of *IL-25* in mast cells, Th2 cells and nuocytes[6–7]. Rather important, nuocytes are helper cells [8]without any lymphocyte markers that express inducible co-stimulator (*ICOS*), *T1/ST2* (IL-33-receptor), *IL-17BR*(*IL-25* receptor), and *IL-7Rα* [9]. They moreover exist in human and mouse livers, gust, and lungs, and can induce tissue repair and Th2 immune responses in airway inflammation [10]. Nuocyte expansions have been demonstrated to act in response to *IL-25* and *IL-33 in vivo*, and were shown to be an early source of IL-13 during a helminth infection with *Nippostrongylusbrasiliensis*[9]. However, the mechanisms underlying *IL-25* in nuocyte activities remain understudied.

Based on previous findings, we hypothesized that *IL-25* in asthma could promote the activation of nuocytes, and then in turn induce a large amount of *IL-5* and *IL-13* to enhance Th2 cytokine responses. In this study, the mice model with OVA was first generated. The expression of both *IL-25* and Th2-type inflammation in bronchoalveolar lavage fluid (BALF) and in lung tissues were then valuated throughout this process. Finally, the asthmatic mice were treated with the *IL-25* antibody in order to observe the inflammatory cell infiltration as well as the inflammatory cytokine secretion.

## Materials and Method

### Animals

The BALB/c mice (n = 45, weight 25±5g, female) were obtained from the Animal Centre of Shandong University, in the Shandong Province of China. The mice were randomly divided into three groups: an asthma group (n = 15), control group (n = 15) and anti-*IL-25* group (n = 15). Chloral hydrate was used for anesthesia. All animal experiments were undertaken with the approval of the Institutional Animal Care and Use Committee of Shandong University [11].

### Model Generation

Considering that the symptoms and pathologic features of the airway inflammation of mice is similar to that of human asthma, an Ovalbumin (OVA) mice model was chosen for our experiments. Mice in both the asthma group and the anti-*IL-25* group were sensitized with 100 μg OVA in 1 mg aluminum hydroxide (Sigma), in a total volume of 0.25ml of PBS twice, on both the 1st and 8th day. They were then challenged with OVA aerosols (1% in PBS) for 30 min per day for 7 consecutive days. The control mice were sensitized with PBS instead of OVA.

In the anti-*IL-25*-treated experiment, mice were given anti-*IL-25* (0.5 mg per mouse) by intranasal before each challenge. Through this experimental design, we expected to see the difference of results between the asthma group and anti-*IL-25* group, particularly when compared to the control. During the experiment, only two mice died due to the sensitized agent–ovalbumin (OVA). Although the mortality rate (4.2%) was observed, the results in this study still showed significant difference within the standard deviation, when this is fully considered in the statistical analysis.

### BALF Collection and Analysis on Inflammatory Cytokines

Collection of BALF was performed with PBS lavage four times (0.5 ml each time) through the cannulated trachea 24 h after the last challenge. A total of 2 ml of BALF was collected from each mouse [9]. The supernatant was then decanted and stored at −80°C for further analysis. The cellular influx in the BALF was examined by means of a cell counting plate. The number of eosinophils was counted by utilizing Wright's staining. The levels of *IL-25*, *IL-5*, and *IL-13* were investigated by enzyme linked immune-sorbent assay (ELISA) kits.

### Histology

The lungs were excised from the chest cavity and fixed with a 10% neutral buffered formalin. Lungs were then embedded in paraffin, sectioned, and stained with hematoxylin and eosin (H&E) [12]. All images were developed at ×40.

### Reverse transcription PCR

The total RNA was isolated from the lung tissues (n = 15 mice for each group) using TRIZOL Reagent (Invitrogen, Carlsbad, California, USA), while cDNA was generated via reverse transcription reaction with hexamer and Superscript II RT (Invitrogen).

All primers were designed and obtained from Takara Biotechnology Co.Ltd (Dalian) (Table 1). PCR was carried out in a 25 μl (final volume) containing the following: 5.0 μl of 5× PCR buffer, 1.0 μl of 10 μmol/L primer (both forward and reverse), 0.15 μl of Taq DNA polymerase, 5.0 μl of 1 μg/μlcDNA template, and 13.85 μl of 0.1% diethylpyrocarbonate-treated water. The PCR conditions for IL-25 and GAPDH were performed as follows: initial denaturation at 95°C for 60 seconds, followed by 37 cycles of denaturation at 95°C for 5 seconds, annealing at 55°C for 120 seconds, and extension at 72°C for 180 seconds. The PCR conditions for IL-25 and GAPDH were as follows: initial denaturation at 94°C for 30 seconds, followed by 40 cycles of denaturation at 95°C for 5 seconds, annealing at 56°C for 30 seconds, and extension at 72°C for 60 seconds. PCR products were separated on 2% agarose gels. The resulting bands were analyzed using an ABI 7900 Imaging System (UVP, LLC Upland, California, USA). GAPDH mRNA levels were used as an internal quantitative control, and the level of each target gene transcript was normalized with GAPDH mRNA levels.

**Table 1:** PCR primer sequences.

Gene	Sequence	Size (bp)
mIL-25		192
Forward	5′-CAGCAAAGAGCAAGAACC-3′	
Reverse	5′-CCCTGTCCAACTCATAGC-3′	
mGAPDH		131
Forward	5′-AACAGGCGTCCCTTTCCGA-3′	
Reverse	5′-GCCCAAGATGCCCTTCAGT-3	

Note: All sequences are in the 5' to 3' orientation.

### Western blot analysis

The tissues used in western blotting analysis were cultured from the lungs of the mice. Whole proteins from the lung tissues were reconstituted in an ice-cold RIPA buffer containing phenylmethanesulfonyl fluoride (PMSF, 1 mM) and protease inhibitors (1:100 dilution; Sigma-Aldrich). The entire tissue was then microdissected and frozen. For total protein extraction, individual cell samples were equalized, and then homogenized with an ice-cold lysis buffer containing 0.5M Tris–HCl, pH 7.4, 1.5M NaCl, 2.5% deoxycholic acid, 10%NP-40,10mM EDTA, and protease inhibitors (Millipore, Bedford, MA). The homogenates were centrifuged at 120,00 rpm for 30min at 4°C. The supernatants were harvested and used for western blotting analysis.

Protein concentrations were determined using the BCA (Beyond) assay. Sample proteins were diluted in 4×lodding buffer solution and boiled at 96°C for 5 min. A 30μg aliquot of (or equal proportion of) concentrated supernatant was subjected to sodium dodecyl sulfate/polyacrylamidegel electrophoresis (SDS-PAGE Bio-Rad, Hercules, CA, USA), and then transferred to nitrocellulose/PVDF membrane following the standard method. Non-specific binding was inhibited through incubation in 5% nonfat dried milk in a 100ml phosphate-buffered saline containing 0.05% Tween-20 (PBST) for 1h at 37°C in a covered container.

Immunoblotting was performed by incubating membranes with rabbit polyclonal *IL-25* antibody (1:500), diluted in 5% bovine serum albumin (BSA) at 4°C overnight. After washing for three times with PBST, the membranes were incubated with secondary goat anti-rabbit IgG-horseradish peroxidase antibodies (1:6000, Santa Cruz), diluted in TBST at 37°C for 1.5h. Finally, the membranes were washed with TBST. Protein bands were detected by the enhanced chemiluminescence method (ECL kit, Amersham, UK). The band intensity was quantified using the Image-Pro Plus image analysis software, and the mean gray value of each band was used for further statistical analysis. The relative levels of *IL-25* were normalized to the goat monoclonal GAPDH (1:3000, Bioworld, USA) band intensity.

### Flow Cytometry analysis

BALF single-cell suspensions were kept in a florescence-activated cell sorting (FACS) EDTA buffer (PBS, 0.5% bovine serum albumin, 5 mM EDTA, 0.1% azide) until immune-fluorescent labeling. Single-cell suspensions were pre-incubated with *IL-33* receptor–blocking antibody (anti-*T1/ST2*, clone 2.4G2), in order to reduce nonspecific binding. Biotinylated anti-*ICOS* (BD Bioscience) monoclonal antibody was used to identify mouse nuocytes. Cells were washed twice with FACS buffer and then analyzed.

### Statistical Analysis

All data was represented as mean ± standard deviation (SD). Both the levels of cytokines and the quantity of *IL-25* were analyzed by one-way analysis of variance. All test results were considered significant at *P*<0.05.

## Results

### Asthmatic Murine Model

**Fig 1:**
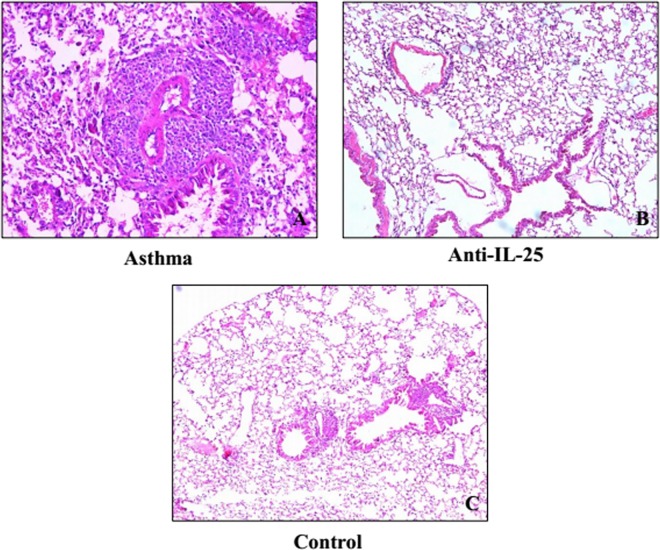
Lung histology. Pathological sections of lung tissues were harvested after 24h, fixed with buffered formalin, and then 5-micrometer sections were analyzed by HE staining. Original magnification, ×40. A. Asthma group. B.Anti-*IL-25* group. C. Control group.

The asthmatic murine model was established in order to detect airway inflammation with the OVA sensitization. Using this, we found an asthmatic phenotype with significantly increased airway eosinophil infiltration, goblet cell metaplasia, and airway hyper-responsiveness. We found this by means of histological analysis. This data indicates that the asthmatic murine model was established successfully (Fig 1).

### Inflammatory Cells

**Fig 2:**
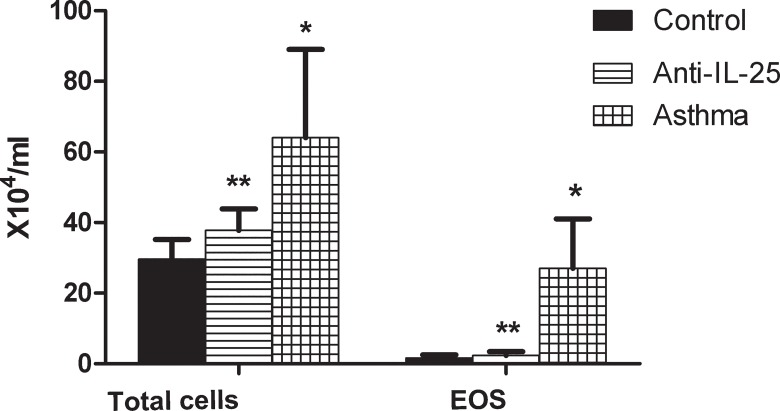
Analysis of BALF total cell numbers and classified inflammatory cells of BALB/c mice, by cell counting plate. Bars depict the mean±SD. * indicates *P* value of less than 0.05; ** indicates *P* value of more than 0.05, compared with control group.

To identify the inflammatory response of the mice at the cellular level, we examined both total cells counts and eosinophils (EOSs) in the BALF, by means of a cell counting plate (Table 2 and Fig 2). Mice exposed to OVA in both groups (asthma and anti-*IL-25*) demonstrated a significant increase in the number of total cells (*P*< 0.05) and eosinophils (*P*< 0.05), particularly compared with those of the other groups (Fig 2). Moreover, in the anti-*IL-25* groups, the eosinophil number decreased significantly (2.36±1.13×10^4^/ml) particularly compared to the total cell number (37.81±6.08×10^4^/ml) (p<0.05). This decrease is similar to that found in the control group (1.64±0.87×10^4^/ml of EOS versus 29.64±5.53×10^4^/ml of total cells). However, this significant variation of cell numbers didn’t occur in asthma group. This suggests that IL-25 activity induced the accumulation of eosinophils and neutrophils.

**Table 2:** Inflammation Responses in the Airway.

Group	N	Total cells count (×10^4^/ml)	EOS Absolute count (×10^4^/ml)	EOS percentage(%)
**Asthma**	**15**	63.12±28.59*	27.47±14.53*	50.6±3.29*
**Anti-IL-25**	**15**	37.81±6.08**	2.36±1.13**	4.83±1.73**
**Control**	**15**	**29.64±5.53**	**1.64±0.87**	**4.26±1.58**

EOS: eosinophil

EOS percentage = EOS absolute / total cell count

EOS Absolute count = total cell count × EOS percentage

* indicates *P* value of less than 0.05, compared with control group

** indicates *P* value of less than 0.05, compared with control group.

### Inflammatory Cytokines

**Fig 3:**
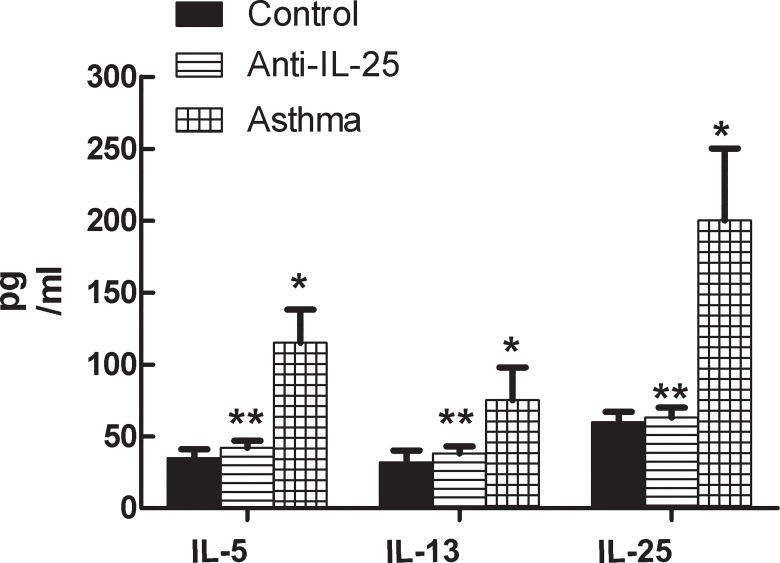
Analysis of inflammatory cytokines in BALF by ELISA. Bars depict the mean±SD. * indicates *P* value of less than 0.05; ** indicates *P* value of more than 0.05, compared with control group.

*IL-25* has been reported as a key cytokine that promotes Th2 immunity responses and enhances the production of other cytokines. To investigate the correlation among the mediators (*IL-25*, *IL-5* and *IL-13*) of Th2 asthmatic inflammation, we detected their expressions in the BALF. Mice exposed to OVA displayed significant hyper-responsiveness (AHR) and increase of inflammatory cells. These are both features of asthma. Moreover, we found a much higher secretion of *IL-5*, *IL-13* and *IL-25* in the asthma group (*P*< 0.05), while no significant changes in the anti-*IL-25* group were observed (*P*> 0.05, Table 3 and Fig 3). This demonstrates that the administration of the anti-*IL-25* antibody inhibited the signaling pathway used to produce *IL-5* and *IL-13*. Therefore, the positive correlation among *IL-25*, *IL-5* and *IL-13* indicates that *IL-25* plays a role in the increase of Th2-type cytokines (*IL-5* and *IL-13*) that induces asthmatic airway inflammation and promotes Th2-type adaptive immune responses.

**Table 3:** Expression of *IL-25*, *IL-5* and *IL-13* by ELISA.

Group	N	IL-25(Pg/ml)	IL-5(Pg/ml)	IL-13(Pg/ml)
**Asthma**	**15**	201.35±47.23*	115.73±13.68*	89.71±7.82*
**Anti-IL-25**	**15**	74.84±25.37**	39.48±5.71**	35.22±4.23**
**Control**	**15**	**68.79±19.26**	**32.17±5.38**	**30.91±3.09**

* indicates *P* value of less than 0.05, compared with control group

** indicates *P* value of less than 0.05, compared with control group.

### Over-expression of IL-25 in OVA-Induced Mice

**Fig 4:**
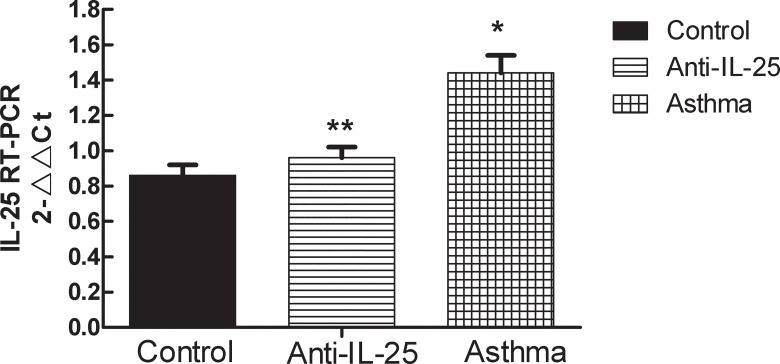
The expression of *IL-25* mRNA in lung tissue was detected by Comparative Ct value. Results are expressed as mean±SD. * indicates *P* value of less than 0.05; ** indicates *P* value of more than 0.05, compared with control group.

**Fig 5:**
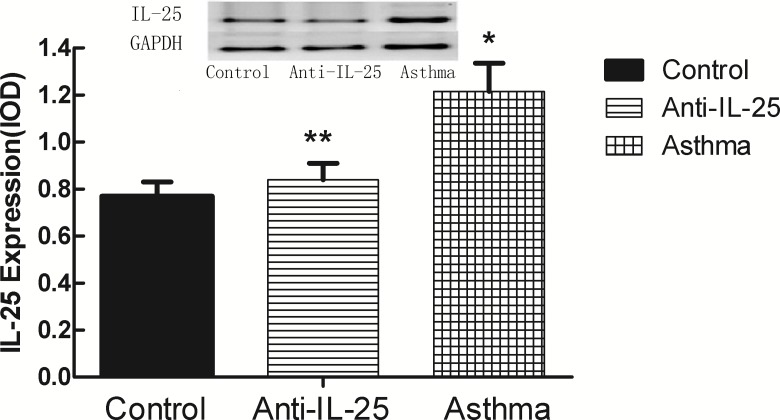
The expression of *IL-25* protein in lungs by means of western blot analysis. The tracheal explants of mice extracted to perform western blot analysis. A. *IL-25* and *GAPDH* mRNA expression profile. B. Results of A were quantified and graphed by densitometry using the Image-Pro Plus software. Results are expressed as mean±SD. * indicates *P* value of less than 0.05; ** indicates *P* value of more than 0.05, compared with control group.

To further confirm the expression pattern of the *IL-25* gene in the OVA-induced asthma mice, we detected the gene’s expressions in the mRNA by means of quantitative real-time PCR, and in the protein by western blot, respectively. Results show that the expression of *IL-25* in both mRNA (Table 4, Fig 4) and protein (Fig 5) exposed to OVA were much higher in the asthma groups than in other groups. The expression in the anti-*IL-25* group (both mRNA and protein) have no significant change, particularly when compared to the control (*P*> 0.05, Fig 4 and Fig 5). This demonstrates that the expression of *IL-25* is significantly up-regulated in OVA-induced asthmatic mice.

**Table 4:** Quantitative Detection of *IL-25* mRNA in lung tissue.

Group	N	GAPDH-Ct(Ct-G)	IL-25-Ct(Ct-IL-25)	2-ΔΔCt
**Asthma**	**15**	16.84±0.93*	21.38±6.02*	1.45±0.08*
**Anti-IL-25**	**15**	13.92±0.52**	16.73±1.02**	0.96±0.06**
**Control**	**15**	**13.26±0.49**	**15.74±0.86**	**0.88±0.05**

* indicates *P* value of less than 0.05, compared with control group

** indicates *P* value of less than 0.05, compared with control group.

### *ICOS* and *T1/ST2* expressions in OVA-Induced Mice

The innate immune system is a non-specific defensive mechanism. It consists of cells and proteins that are ready to defend against pathogenic infection. Th2-mediated immune response has been reported to play a crucial role in the pathophysiology of asthma through the induction of Th2 type cytokine production, by means of *IL-25* [13–14]. Important cells in the immune system, nuocytes are usually defined by various cell surface markers: *ICOS*, (a member of the *CD28/CTLA-4* family) and receptors for cytokine *IL-33* (*T1/ST2*) [15].

**Fig 6:**
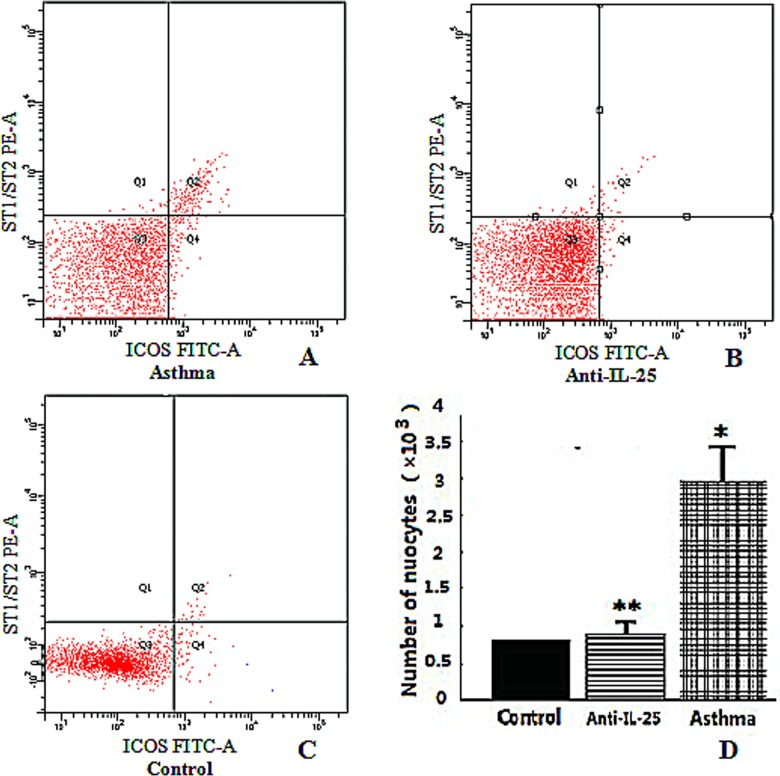
The number of nuocytes expressed in BALF by means flow cytometry analysis. The numbers in A, B, and C represent the absolute numbers of detected cells in the boxes. A. The levels of *ICOS* and *T1/ST2* in nuocytes were significantly up-regulated in the asthma group. B. The levels of *ICOS* and *T1/ST2* in nuocytes were not significantly changed in the anti-IL-25 group. C. The levels of *ICOS* and *T1/ST2* in nuocytes were not significantly changed in the control group. D. The number of nuocytes. Result are expressed as mean±SD. * indicates *P* value of less than 0.05; ** indicates *P* value of more than 0.05, compared with control group.

In this study, to identify the detailed role of *IL-25* in promoting innate immune responses in asthmatic airway inflammation, the expression levels of *ICOS* and *T1/ST2* on nuocytes were detected by means of flow cytometry. We found that the levels of *ICOS* and *T1/ST2* on nuocytes were significantly up-regulated in BALF of OVA-induced mice, particularly when compared with the control group (*P*< 0.05, Fig 6). However, the levels were down-regulated when treated by intranasal anti-*IL-25*. There is no difference between the anti-*IL-25*-treated group and the control group. Thus, we see nuocytes are involved in *IL-25* signaling and, moreover, interact with a wide range of mediators. This could trigger the activation of Th2-type immune responses (Fig 6).

## Discussion

### *IL-25* induces the production of *IL-5* and *IL-13*

Asthma is a chronic airway inflammatory disease. It occurs with a predominant Th2 immunity, and is a consequence of inappropriate immunologic responses to environmental allergens. Infiltration of eosinophils and other cells, excessive mucus production, and airway hyper-reactivity all induces airway narrowing and generates significant morbidity [1].

As a member of the *IL-17* family, interleukin-25 promoted Th2 immune responses [16]. But other *IL-17* family cytokines have similar reactions to Th1 inflammatory cytokines [17]. Importantly, recent studies have reported that the expression of *IL-25* is associated with asthmatic disease in both human and mice’s lung epithelial cell lines [18]. This indicates that *IL-25* is an important initiator of type-2 responses. However, other studies have questioned whether the type of cells that produce *IL-25* is not only in Th2 cells [4], but also in eosinophils and basophils [19].

More recently, it has been demonstrated that *IL-25* mRNA is up-regulated in the gut after a *Nippostrongylusbrasiliensis* infection, or in the lungs in response to *Aspergillusfumigatus*[20]. In addition, some studies have detected the expression of *IL-25* in mast cells, Th2 cells, NKT cells, and nuocytes[21–22].

In our study, the over-expression of *IL-25* was found in the OVA murine model, consistent with most previous conclusions [23–24]. Furthermore, mice were found to be able to induce a large amount of *IL-5* and *IL-13* with the treatment of *IL-25*. This indicates that *IL-25* promotes Th2 immunity response through the recruitment of *IL-5* and *IL-13*.

### The expression of IL-25 induces nuocytes

As the antigen-presenting cells that bridge between innate and adaptive immunity, nuocytes induce tissue repair and Th2 immune responses [13]. From the expression profile of *IL-25*, Th2-type inflammation in BALF, and lung tissues in our study, *IL-25* was observed to be significantly over-expressed in both lung tissues and the BALF. Moreover, the expression of *ICOS* and *T1/ST2* in BALF was also increased on the nuocytes of asthmatic mice, but was down-regulated in mice treated by intranasal application of anti-*IL-25*. Furthermore, when the asthmatic mice were treated with the *IL-25* antibody, the inflammatory cell infiltration and the inflammatory cytokine secretion were significantly decreased. This represents a positive correlation between *IL-25* and nuocytes, implying that *IL-25* expression might be involved in a series of asthmatic immune responses, and play an important role in nuocytes activation (that maintains Th2 central memory and sustains asthmatic inflammation).

There are some studies that demonstrate the direct correlations between production of *IL-5* and *IL-13*, and nuocytes promoted by *IL-25*[9] or *IL-33* [25]. Overall, the emerging picture in innate type-2 immunity is one of multiple cell types (including NHCs, nuocytes, Ih2 cells, MPP type-2 cells, basophils, mast cells, NKT cells, and eosinophils) with distinct but overlapping roles. Most of these cells respond to one or more of cytokines *IL-25*, *IL-33*, and *TSLP*. More importantly, most have the capacity for significant production of type-2 cytokines [9]. Thus, we believe that the activities of Th2 cytokines *IL-5* and *IL-13* promoted by *IL-25* in asthmatic mice are intermediated by nuocytes, which in turn might accumulate *ICOS* and *T1/ST2*.

### Treatment of the *IL-25* antibody, and airway remodeling

Airway remodeling is defined as the structural changes occurring in both large and small airways [26]. Airway remodeling is usually featured by the increase of airway wall thickness, allergic airway inflammation, epithelial cell alterations (cell shedding, ciliated cell loss, and goblet cell hyperplasia), subepithelial fibrosis, the increase of airway smooth muscle mass, and cronchial neovascularization [27]. Clinically, the understanding of airway remodeling pathogenesis is poor, which leads to lackingtherapeutic targets in clinic trials. There are few studies that have reported that *IL-25* drives airway remodeling [28–29]. This is consistent to our findings.

Our study also indicates that *IL-25* activates nuocytes with high expression of *ICOS* and *T1/ST2*, and interacts with other inflammatory mediators. This initiates the ensuing adaptive Th2-type immune responses in asthmatic mice. However, when asthmatic mice were given *IL-25* antibodies, airway inflammation was inhibited by the reduced infiltration of inflammatory cells and cytokines. The mice receiving the *IL-25* antibody suffered from a significant decrease in the expression of *ICOS* and *T1/ST2* on their nuocytes. This implies that the *IL-25* antibody might inhibit the activation of nuocytes, so as to decrease the Th2-related mediator production.

Therefore, our study indicates that *IL-25* might be a potential clinical target for the treatment of airway remodeling in asthma. This moreover supports the conclusions in previous studies about airway remodeling[30–31]. The *IL-25* antibody can be considered a good therapeutic candidate for the management of asthmatic airway inflammation.

## Conclusions

In summary, this study revealed that *IL-25* was a potential active cytokine in the OVA-induced asthmatic model. *IL-25* induced strong type-2 adaptive immune responses. In addition to its direct effect, *IL-25* could also induce a strong expression of both *IL-5* and *IL-13* via the nuocytes. Moreover, *IL-25* might be a potential clinical target for the treatment of airway remodeling in asthma, and its antibody maybe considered a good therapeutic candidate for the management of asthmatic airway inflammation.
